# Inflammatory bowel disease-associated *Escherichia coli* strain LF82 in the damage of gut and cognition of honeybees

**DOI:** 10.3389/fcimb.2022.983169

**Published:** 2022-08-25

**Authors:** Ruqi Chang, Jieteng Chen, Zhaopeng Zhong, Yiyuan Li, Kaichun Wu, Hao Zheng, Yunsheng Yang

**Affiliations:** ^1^ Medical College of Nankai University, Tianjin, China; ^2^ Beijing Advanced Innovation Center for Food Nutrition and Human Health, College of Food Science and Nutritional Engineering, China Agricultural University, Beijing, China; ^3^ Microbiota Division, Department of Gastroenterology and Hepatology, The First Medical Center, Chinese PLA General Hospital, Beijing, China; ^4^ Xijing Digestive Hospital, Xian, China

**Keywords:** *Apis mellifera*, LF82, IBD, cognitive impairment, microbiota-gut-brain axis

## Abstract

Patients with inflammatory bowel disease (IBD) are often accompanied with some cognitive impairment, but the mechanism is unclear. By orally exposing honeybees (*Apis mellifera*) to IBD-associated *Escherichia coli* LF82 (LF82), and non-pathogenic *Escherichia coli* MG1655 (MG1655) as the normal strain, we investigated whether and how LF82 induces enteritis-like manifestations and cognitive behavioral modifications in honeybees using multiparametric analysis. LF82 significantly increased gut permeability, impaired learning and memory ability in olfactory proboscis extension response conditioning, and shortened the lifespan of honeybees. Compared to MG1655, LF82 reduced the levels of tryptophan metabolism pathway substances in the honeybee gut. LF82 also upregulated genes involved in immune and apoptosis-related pathways and downregulated genes involved in G protein-coupled receptors in the honeybee brain. In conclusion, LF82 can induce enteritis-like manifestations and cognition impairment through gut metabolites and brain transcriptome alteration in honeybees. Honeybees can serve as a novel potential model to study the microbiota-gut-brain interaction in IBD condition.

## Introduction

Inflammatory bowel disease (IBD) is a complex group of diseases that causes chronic inflammation of the gastrointestinal tract, including ulcerative colitis and Crohn’s disease (CD). Clinical studies have shown that the prevalence of cognitive impairment is significantly higher in patients with IBD (Dancey et al., 2009; Clarke et al., 2020; Sharma et al., 2021).

Although the etiology of IBD is not fully understood, it is well-known that the gut microbiota is involved in the development and maintenance of IBD (Ni et al., 2017; Glassner et al., 2020; Lee and Chang, 2021). Many clinical independent groups have reported that mucosa‐associated *Escherichia coli* is increased in patients with IBD (Darfeuille-Michaud et al., 2004; Martin et al., 2004; Kotlowski et al., 2007; Martinez-Medina et al., 2009). Additionally, the adherent-invasive *E. coli* (AIEC) has been implicated in the pathogenesis of CD (Chervy et al., 2020). As one of the AIEC reference strains, *E. coli* LF82 has been isolated from the ileal mucosa of patients with CD (Darfeuille-Michaud et al., 1998; Boudeau et al., 1999; Martinez-Medina et al., 2009; Palmela et al., 2018). In contrast to commensal *E. coli*, strain LF82 damages the host intestinal epithelium by inducing reactive oxygen species production, destroying intestinal epithelial cell mitochondria, and inhibiting the activation of the *NF-κB* signaling pathway in macrophages (Elatrech et al., 2015; Sevrin et al., 2020; Mancini et al., 2021). As mentioned above, most studies have focused on the damage of the intestinal mucosa (Carriere et al., 2016), and there are still relatively few studies on the damage and mechanism of extra-intestinal such as cognition.

Over the past decades, there has been growing evidence that the gut microbiota is involved in brain disorders (Cryan et al., 2019). Gut microbes interact with the brain through the gut-brain axis to co-regulate immune, metabolic, and nervous system development and function (Morais et al., 2021). We now understand some of the pathophysiological consequences of an aberrant gut-brain network, including exacerbated disturbances in gut inflammation, altered responses to acute and chronic stress, and altered behavioral states (Osadchiy et al., 2019). However, the pathways by which 1) gut microbes communicate with the nervous system and 2) the host responds to specific gut microbes remain unclear.

Studies observing the cognition of animals following infection due to enteric bacterial pathogens offer a more accurate representation of the human condition in certain instances (Goehler et al., 2008; Bercik et al., 2010). The honeybee (*Apis mellifera*) has a simple and specific gut microbiota, which makes it advantageous as a model system for microbiota research (Kwong and Moran, 2016; Zheng et al., 2018). Furthermore, the honeybee is a model organism for the study of learning and memory formation. A set of established methods are available for quantifying complex bee behaviors, such as associative learning and memory, sensory responses, and hive behavior observations (Menzel, 2009). Therefore, it is feasible to study interactions between gut microbiota and the brain using an intestinal infection model of honeybees.

MG1655 is a non-invasive, non-pathogenic, human symbiotic *E. coli* K-12 strain, which is commonly used as a non-pathogenic negative control strain in pathogenic bacteria research (Blattner et al., 1997; de Sousa Figueiredo et al., 2021).

In this study, we used non-pathogenic *E. coli* MG1655 (MG1655) and the IBD-associated *E. coli* LF82 to study whether *E. coli* LF82 infection impairs the ability of learning and memory in honeybees and explore how *E. coli* LF82 might cause any cognitive differences in normal, healthy honeybees. Furthermore, we focused on changes in gut metabolites and brain transcriptome in honeybees.

## Materials and methods

### Bacterial cultures and exposure

*E. coli* LF82 was isolated from the ileal lesion of a CD patient. The LF82 used in this study was kindly donated by Dr. Kaichun Wu (Xijing Digestive Hospital, Xian, China), and has been previously identified (Boudeau et al., 1999). MG1655 was purchased from Biobw (Beijing, China).

Both LF82 and MG1655 were kept as an aliquoted stock in 25% glycerol at -80°C. The stock solution was removed from the freezer, streaked on Luria-Bertani (LB) agar, and incubated overnight at 37°C. After 12 hours, approximately five single colonies were selected and streaked on LB medium for amplification. About 24–48 h later, appropriate amounts of colonies were scraped and dissolved in 50% sucrose solution at a final OD600nm of 1, to replicate an appropriate diet for the honeybees.

### Subjects and groups

**Figure 1:**
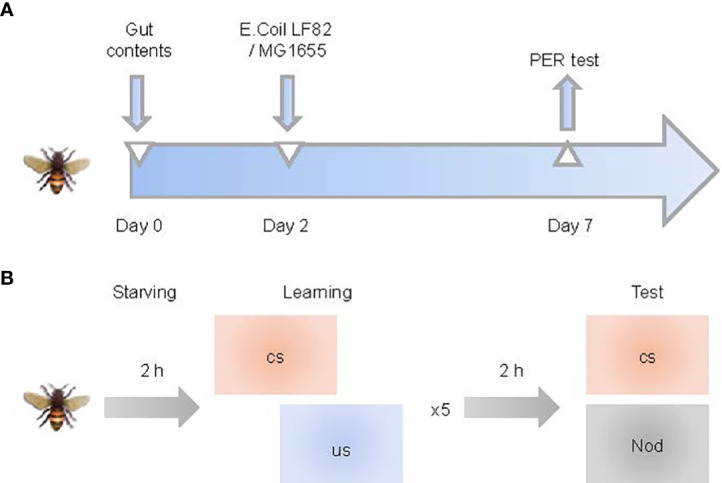
Study pipeline. **(A)** Schematic illustration of experimental design. Honeybees (0–1 day old) were fed with sucrose water-mixed communities from whole-gut contents and sterile pollen for two days and then orally exposed with *Escherichia coli* for five days. **(B)** Training and memory protocol. Bees were starved 2 h before five trials of olfactory proboscis extension response (PER) conditioning. Memory was tested 1 h after conditioning by presenting the conditioned stimulus (CS) and a novel odor (NOd).

Honeybees (worker bees) were collected from brood frames in Shunyi County of Beijing in July 2021. All bees used in the study came from the same colony. Honeybees 0–1 day old were collected in sterile cages, kept at 35°C for 2 days, and fed with filter-sterilized 1:1 (w/v) sucrose solution containing normal honey bee intestinal homogenate, to normalize the gut microbiota of bees (Zheng et al., 2017). Bees were then divided into three groups: 1) control, 2) MG1655-colonized (MG1655 group) and 3) LF82-colonized (LF82 group). Each group had three replicating cup cages containing approximately 20 honeybees in each cup. For the control group, honeybees were fed with sterile pollen and filter-sterilized 1:1 (w/v) sucrose. For the MG1655 or LF82 group, *E. coli* MG1655 or LF82 were mixed in filter-sterilized 1:1 (w/v) sucrose at a final OD600nm of 1. A PER (proboscis extension response) test was conducted 5 days after initial infection when the honeybees were 7–8 days old (**Figure 1A**). The gut and brain samples were collected from different bee individuals after the PER test., which were from the same period and intervened using the methods previously described.

### Survival assays

We monitored and recorded the number of deceased bees in each cup cage every day. The initial number of bees in each cup was recorded at the time of bacterial exposure. Survival curves were generated using GraphPad Prism version 9 (GraphPad Software, San Diego, California, USA). Multiple comparison tests were performed using the log-rank test.

### Smurf assays

We adapted a non-invasive “Smurf” assay in a *Drosophila* model to honeybees during exposure to *E. coli* to monitor possible gut leakage (Wong et al., 2009). Non-toxic blue dye was dissolved in 50% sucrose solution to prepare a final concentration of 1% blue solution, and the bees were fed freely and continuously. We classified bees with blue hemolymph or blue entire abdomen as Smurf (+), as opposed to a well-defined blue linear outline (gut) or nothing in healthy individuals.

### Gut hematoxylin-eosin staining

Dissected guts were fixed overnight in 8% w/v paraformaldehyde. After fixation, the guts were rinsed with phosphate-buffered saline at room temperature. The tissues were then dehydrated into xylene through an ethanol series. The dehydrated tissues were stored in xylene + paraffin in an oven at 60°C. Finally, the tissues were embedded in paraffin and stored at 0°C for at least 24 h. Five-μm sections obtained from each paraffin block were immersed in xylene and alcohol, stained with hematoxylin for 5 min followed by 1% acid ethanol (1% HCl in 70% ethanol). Samples were then rinsed in distilled water, stained with eosin for 3 min, and re-immersed in alcohol and xylene. Slides were mounted using a synthetic resin, and the morphological structure of the gut tissue was observed under the microscope (Leica, Wetzlar, Germany).

### Gut DNA extraction and 16S rRNA sequencing

DNA was extracted from the dissected gut using cetyltrimethylammonium bromide (CTAB) and bead-beating: the dissected gut was placed in a tube containing approximately 0.5 mL of 0.1-mm silica zirconia beads (BioSpec Products, Bartlesville, OK, USA), 728 μL CTAB, 2 μL 2-mercaptoethanol (Sigma-Aldrich, St. Louis, MO, USA) and 20 μL of 20 mg/mL proteinase K (Sigma-Aldrich, St Louis, MO, USA). These tubes were then stirred in a multi-sample bead beater (BioSpec Products, Bartlesville, OK, USA) at full speed for 2 min, placed on ice for 1 min, and bead agitated for 2 min. Sample tubes were then incubated at 56°C overnight. RNase A (Sigma-Aldrich, St Louis, MO, USA) was added to the tubes (5 μL), which were vortexed briefly using a lab vortex mixer (Shanghai Medical University Instrument Factory, Shanghai, China) and placed at 37°C for 1 h. The samples were combined with 0.75 mL phenol-chloroform-isoamyl alcohol (25:24:1), shaken for 30 s, and placed on ice for at least 2 min. The sample tubes were centrifuged at full speed for 15 min at 4°C, and the aqueous phase was alcohol precipitated, washed, air-dried, and resuspended in 50 μL nuclease-free water.

The 16S rRNA genes of V3–V4 regions were amplified using specific primers (515F:50- CCTAYGGGRBGCASCAG -30 and 806R: 50-GGACTACNNGGGTATCTAAT-30). All polymerase chain reaction (PCR) reactions were performed using 15 μL Phusion® High-Fidelity PCR Master Mix (New England Biolabs, Ipswich, USA), and the PCR products were detected by electrophoresis and purified with QIAquick Gel Extraction Kit (Qiagen, Hilden, Germany).

High-throughput sequencing was performed at Novogene Bioinformatics Technology Co. Ltd., Beijing, China, on an Illumina NovaSeq6000 platform (Illumina, San Diego, CA, USA). Sequencing libraries were generated using a NEBNext® Ultra™ II DNA Library Prep Kit (Cat No. E7645). Subsequently, 250 bp paired-end reads were generated. The quality of the raw data, as well as the number of sequences and amplicon sequence variants, were evaluated using the Quantitative Insights into Microbial Ecology 2 bioinformatics platform (Bolyen et al., 2019).

### Metabolomic analysis of the gut

Each dissected gut was quickly sampled and stored at −80°C. Six biological replicates from each group were used for untargeted metabolomics analysis. The liquid chromatography-mass spectrometry (LC-MS) analyses were performed using a Vanquish UHPLC system (Thermo Fisher, Waltham, MA, USA) with an Orbitrap Q Exactive HF-X mass spectrometer (Thermo Fisher) in positive and negative ionization modes by Novogene Co., Ltd. (Beijing, China).

Tissues from each sample were ground and homogenized in liquid nitrogen before adding them to prechilled 80% methanol and 0.1% formic acid (500 μL for 100 mg gut). After vortexing, the samples were incubated on ice for 5 min, then centrifuged at 15,000 × g and 4°C for 5 min. The supernatant was diluted in LC-MS grade water to the final concentration of 53% methanol, then transferred to a new tube and centrifuged at 15,000 × g and 4°C for 10 min. Finally, the supernatant was injected into the LC-MS/MS system. Samples were injected onto a Hypesil Gold column (100×2.1 mm, 1.9μm) using a 17-min linear gradient at a flow rate of 0.2mL/min. The eluents for the positive polarity mode were eluent A (0.1% FA in Water) and eluent B (Methanol). The eluents for the negative polarity mode were eluent A (5 mMammonium acetate, pH 9.0) and eluent B (Methanol). Same amounts of supernatant from each processed sample in one pipeline were pooled and used as a quality control sample. The data were acquired using the Xcalibur software (version 4.1; Thermo Fisher, Waltham, MA, USA) and Tune software (version 2.9; Thermo Fisher, Waltham, MA, USA). The raw data files generated by UHPLC-MS/MS were processed using the Compound Discoverer 3.1 (CD3.1, ThermoFisher) to perform peak alignment, peak picking, and quantitation for each metabolite. The normalized data was used to predict the molecular formula based on additive ions, molecular ion peaks and fragment ions. And then peaks were matched with the mzCloud(https://www.mzcloud.org/), mzVaultand MassListdatabase to obtain the accurate qualitative and relative quantitative results. Metabolomics data analysis was performed using the MetaboAnalyst interface (software version 5.0) (Pang et al., 2021).

### Olfactory PER conditioning

Associative olfactory learning and memory experiments were performed according to the standard PER protocols (Menzel, 2009; Matsumoto et al., 2012; Zhang et al, 2021) with modifications as follows (**Figure 1B**): 25 bees were taken from each group 5 days after the *E. coli* exposure. Bees were tied into a sectioned hollow metal tube and starved for 2 h by removing foods. To test the appetitive motivation, we touched each bee’s antenna with 50% sucrose solution (without feeding them). Bees that did not respond to proboscis extension were excluded from the experiment. Finally, there were 20–25 bees left in each group to continue the experiment.

We used nonanol (conditioned stimulus, CS; Sigma-Aldrich, Saint Louis, MO, USA), 50% sucrose solution (unconditioned stimulus, US), and hexanal (novel odor, NOd; Macklin, Shanghai, China) as odor sources. A filter paper impregnated with the odorant in the syringe was used for odor delivery.

At least 25 s before the stimulation, bees were set individually with a ventilation hood (to exhaust the odor) at their back. Individual bees were then subjected to a 4-s odor stimulation (CS) followed by a 3-s sucrose stimulation (US) with a 3-s interstimulus interval (1 s overlap). Bees were trained for five trials with a 10-min interval between trials to associate nonanol odor (CS) with a reward of 50% sucrose solution (US).

Two odors (CS and NOd) were presented to trained bees 1 h after conditioning to test their memory retention. The retention test spanned the same amount of time as the conditioning test; the only difference was the absence of US reward in the retention test. Bees that extended their proboscises only to the smell of nonanol were considered subjects with successful memory.

### Brain tissue collection and RNA-sequencing

Honeybee brains were extracted under a dissecting microscope (Nikon SMZ745T, Japan) and fixed on beeswax using insect needles placed through the thorax. After removal of the head cuticle, the whole brain was placed on a glass slide and later immersed in RNA (Thermo Fisher, Waltham, MA, USA) for gene expression profiling. The dissected brains were frozen and maintained at −80°C.

Total RNA was extracted from individual brains using the Quick-RNA MiniPrep kit (Zymo Research, Irvine, CA, USA). RNA integrity was characterized using the RNA Nano 6000 Assay Kit of the Bioanalyzer 2100 system (Agilent Technologies, CA, USA). High-throughput sequencing was performed on an Illumina NovaSeq platform at Novogene Bioinformatics Technology Co. Ltd., Beijing, China. RNA sequencing libraries were generated using NEBNext Ultra RNA Library Prep Kit for Illumina (New England BioLabs, Ipswich, USA). Clusters were generated using a TruSeq PE Cluster Kit v3-cBot-HS (Illumina, San Diego, CA, USA), and RNA-seq libraries were sequenced on an Illumina NovaSeq platform (Illumina, San Diego, CA, USA). Subsequently, 150 bp paired-end reads were generated. The sequences were assessed and aligned using Burrows-Wheeler Aligner’s Smith-Waterman Alignment (BWA-SA) with default parameters (Li and Durbin, 2010). Gene expression was quantified using HTSeq v0.7.2 (Anders et al., 2014). Differential expression analysis was performed using the DESeq2 package (v1.20.0) (Love et al., 2014). Functional analysis of differentially expressed genes was performed based on KEGG Orthologue markers using cluster profile v3.10.1 (Wu et al., 2021).

### Data Availability

The raw data of the 16S rRNA sequencing and brain transcriptome can be found in the BioProject PRJNA813992 and BioProject PRJNA814736, respectively. The gut metabolomics data can be found in the MetaboLights with the identifier MTBLS4475.

## Results

### LF82 decreased the lifespan of honeybees

**Figure 2:**
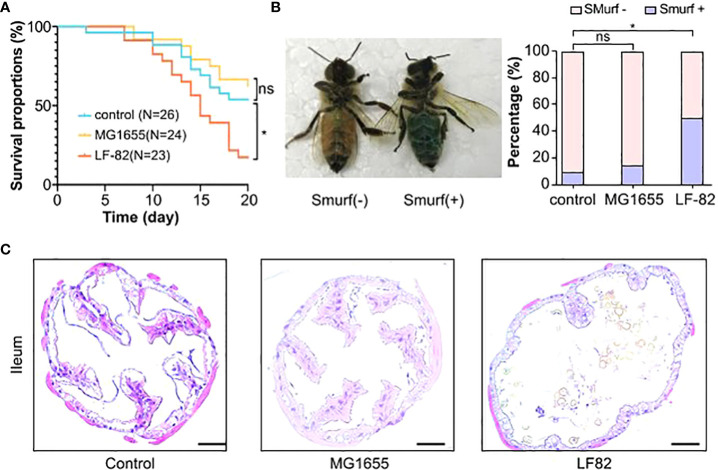
LF82 impacts the gut and decreases the lifespan of the honeybee. **(A)** The Kaplan–Meier survival curves of honeybees in different groups (*p < 0.05; ns, not statistically significant). **(B)** Positive and negative honeybees in the Smurf experiment and the proportion in each group (*p < 0.05). **(C)** Histopathologic evaluation with hematoxylin and eosin staining shows structural disruption in the epithelium of illem of the LF82 group (Bars = 50 μm).

We first investigated if the treatment leads to increased mortality of honeybees (**Figure 2A**). The median survival time of honeybees in the control, MG1655, and LF82 groups were 21.5, 22, and 15 days, respectively. The survival rate significantly decreased in the LF82 group (21.7%) compared to the control (57.7%, *p* < 0.05) and MG1655 groups (67.7%, *p* < 0.05) on the 20th day.

### LF82 induces enteritis-like manifestations in honeybees

We adapted a non-invasive “Smurf” assay from *Drosophila* melanogaster to evaluate the effect of LF82 on intestinal permeability in honeybees (**Figure 2B**). Bees were fed with edible blue dye, which could not be absorbed through their intestines, to observe possible intestinal leakage. LF82 exposure led to a distinct Smurf phenotype that showed a diffuse blue color of the body and hemocoel, unlike the control and MG1655 groups, which exhibited a well-delineated blue digestive tract in the abdomen. In the control, MG1655, and LF82 groups, the proportion of Smurf-positive bees was 10%, 15%, and 50%, respectively. Compared with the control group, the honeybees with leaky gut in the LF82 group were significantly increased (*p* < 0.05, chi-square test). We also stained the ileum (**Figure 2C**) and midgut (**Figure S1**) with hematoxylin-eosin to observe whether the intestinal structure changed. Compared with the control and MG1655 groups, the disappearance of ileum epithelial villi and enlargement of the intestinal lumen was evident in the gut of honeybees in the LF82 group.

### LF82 alters gut metabolites in honeybees

**Figure 3:**
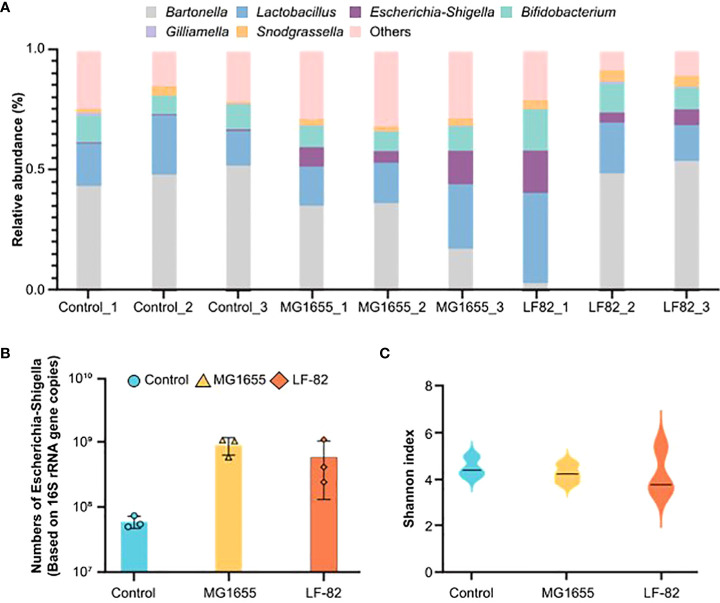
LF82 and MG1655 have no effect on the honeybee gut microbial diversity. **(A)** Relative abundance of core bacteria in the gut of honeybees in the different groups (n = 3). **(B)** Contents of *Escherichia coli* in the gut of honeybees in the three groups (n = 3). **(C)** α-diversity in the different groups (*p*> 0.05).

To explore the underlying mechanisms involved in LF82 on cognition, we first focused on the intestinal tract and detected the gut microbiota and metabolome. The relative content of *E. coli* in the bee gut increased in the MG1655 and LF82 groups, and the absolute content in these two groups was > 10. In the upper right corner label CFU/mL, which was significantly higher than that in the control group (**Figures 3A, B**). Notably, the effect of oral administration of two *E. coli* strains on the honeybee gut microbial diversity was not statistically different (**Figure 3C**). The results indicate that the intestinal symbiotic bacteria of honeybees have a certain protective effect against foreign bacteria.

**Figure 4:**
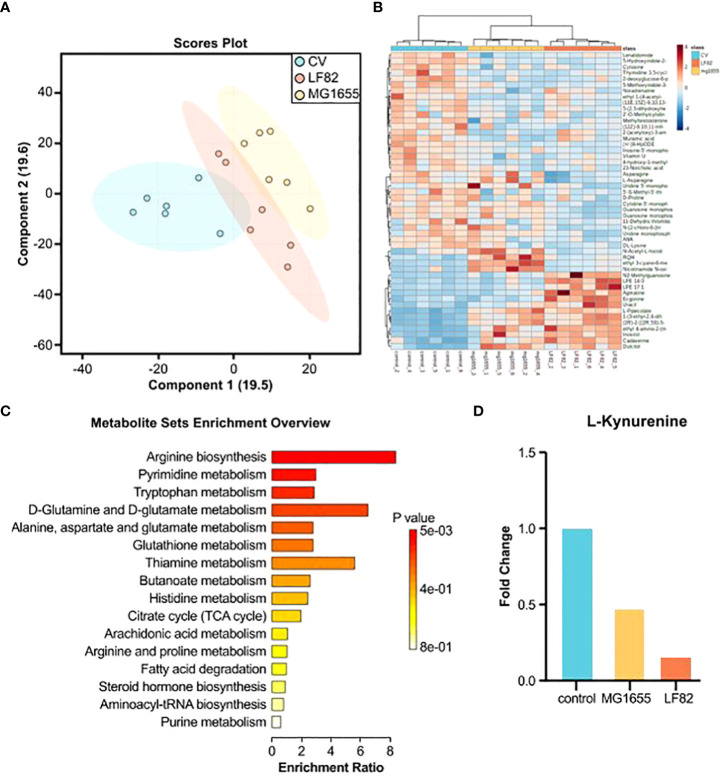
LF82 impacts gut metabolites in the honeybee gut. **(A)** Sparse partial least squares discriminant analysis (PLS-DA) based on all metabolites detected in the gut of honeybees. **(B)** Unsupervised hierarchical clustering heatmap of the 50 metabolites that contribute most to the separation of different groups in the gut samples. **(C)** Down-regulated metabolite KEGG enrichment pathway in the LF82 group. **(D)** The levels of L-kynurenine in each group.

For a more in-depth understanding of the effects of LF82, we compared the level of different metabolites in separate guts of honeybees from the different groups using untargeted metabolomics and gas chromatography/mass spectrometry analysis. The partial least squares discriminant analysis showed that both strains of *E. coli* had significant effects on the gut metabolome (**Figure 4A**). Compared with the control and MG1655 groups, 372 (213 up and 159 down) and 169 (100 up and 69 down) different metabolites from the respective group were identified in the guts of honeybees from the LF82 group (**Figure 4B**). Subsequently, we performed KEGG enrichment analysis for the up- and down-regulated differential metabolites, respectively (**Figures 4C** and **S2A**). Down-regulated metabolites were concentrated in pathways such as arginine biosynthesis as well as pyrimidine and tryptophan metabolism (**Figure 4C**). Among the differential metabolites, the levels of L-kynurenine (**Figure 4D**) and melatonin, which are extensively involved in tryptophan metabolism related to cognition, were significantly reduced in the LF82 group. In addition, we enriched the fecal metabolite database of human disease-related metabolites in the gut of LF82 bees and found that these metabolites are also up-regulated in human IBD and other related diseases (**Figure S2B**).

### LF82 impairs learning ability of honeybees

**Figure 5:**
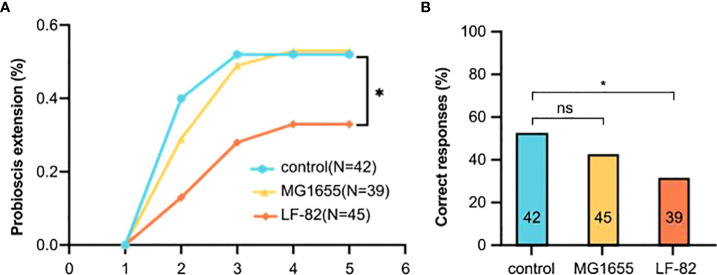
LF82 impairs learning ability of the honeybee. **(A)** Learning curves to a positively rewarded conditioned stimulus (CS). Honeybees in the LF82 group showed impaired ability of learning (*p < 0.05). **(B)** Performance in the memory test is shown as percentage of responses to the CS only; numbers in bars are sample sizes. (*p < 0.05; ns, not statistically significant).

To elucidate the effect of LF82 on cognition (i.e., learning and memory) in honeybees, olfactory proboscis extension response (PER) conditioning and memory tests were conducted after 5 days of *E. coli* treatment. Throughout multiple training trials, more than half the bees in the control and MG1655 groups successfully learned to identify the conditioned stimulus on the third training; accuracy rates of the third round of the control, MG1655, and LF82 groups were 52%, 49%, and 28%, respectively. After the fifth round of learning, the correct rates for the three groups were 52%, 53%, and 33%, respectively. The accuracy rate of honeybees in the LF82 group was much lower than that of the control group (*p* < 0.05, chi-square test) (**Figure 5A**).

In the memory test, 52%, 42%, and 31% of the honeybees in the control, MG1655, and LF82 groups, respectively, were able to correctly identify and respond to the smell. The accuracy rate of honeybees in the LF82 group was lower than that observed in the control group (*p* < 0.05, chi-square test) (**Figure 5B**).

### LF82 induces transcriptomic changes in the honeybee brain

**Figure 6:**
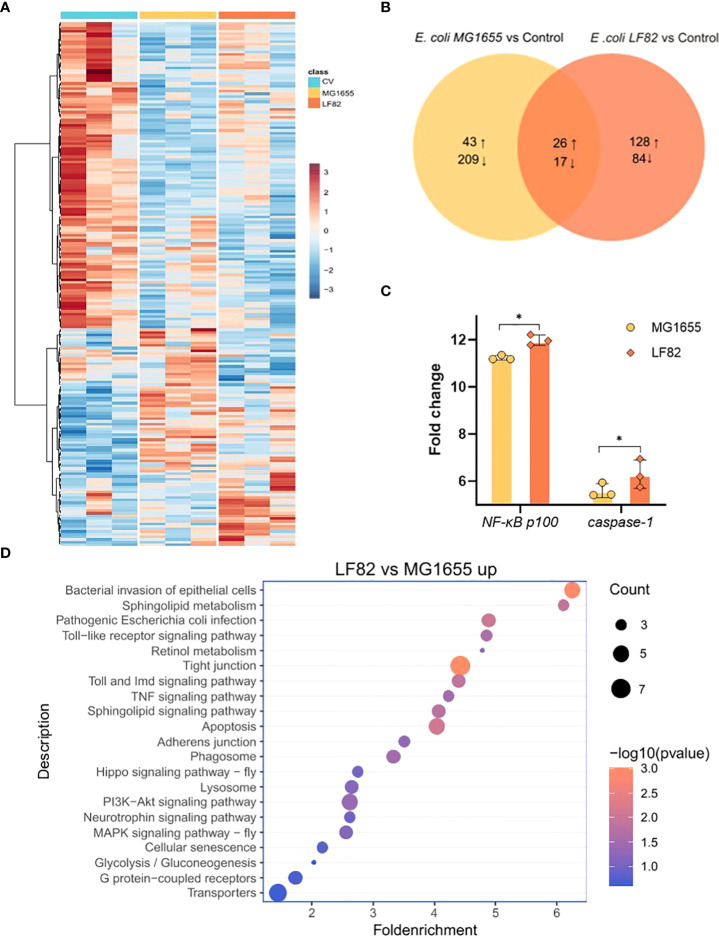
LF82 impacts gene expression in the honeybee brain. **(A)** Heatmap displaying the gene expression among the three different colonization groups. Colors indicate the normalized relative expression (minimum-maximum) for each gene. **(B)** Venn diagram representing the number of significantly differentially expressed genes (DEGs) between groups colonized with *Escherichia coli* LF82 and MG1655, respectively. Numbers show the sum of up- and down-regulated genes. **(C)** Fold change in transcript levels of the *NF-κB* and *caspase-1* genes in the brain of honeybee colonized with MG1655 and LF82. (*p < 0.05) **(D)** Representative enriched KEGG pathways upregulated in the LF82 group compared with the MG1655 group.

To further investigate the molecular mechanisms underlying the effects of LF82 challenge on cognition, we examined transcriptome changes in the honeybee brain. The top 200 significantly differentially-expressed genes were plotted in a heatmap, which showed unique profiles across groups (**Figure 6A**). LF82 colonization altered the expression of 255 host genes (154 upregulated and 101 downregulated). MG1655 colonization altered the expression of 295 host genes (69 upregulated and 226 downregulated). Among the differentially-expressed genes shared by the two *E. coli* groups, 26 genes were up-regulated and 17 genes were down-regulated (**Figure 6B**), which were not significantly enriched in the KEGG pathway (count ≤ 2).

Next, we compared the honeybee brain transcriptome differences between MG1655 and LF82. Compared with the MG1655 group, the LF82 group had 317 up-regulated and 78 down-regulated genes (**Figure S3A**). Gene set enrichment analysis showed that up-regulated differential genes, such as *NF-κB p100* and *caspase-1* (**Figure 6C**), are widely involved in immune- and inflammation-related pathways such as bacterial invasion of epithelial cells, Toll and Imd signaling pathways, apoptosis, and TNF signaling pathways (**Figure 6D**). The down-regulated genes were *NF-κB* inhibitor *cactus, Or33*, and *Or52*, which are mainly enriched in the cAMP signaling pathway and G protein-coupled receptors pathways (**Figure S3B**).

## Discussion

Patients with IBD present with certain neuropsychiatric disorders such as anxiety and depression, and cognitive decline (Dancey et al., 2009; Yuan et al., 2021), the severity of which are positively correlated with the severity of the disease (Clarke et al., 2020); however, the mechanism of this correlation remains unclear. It is well known that pathogenic *E. coli* plays an important role in the development of IBD (Darfeuille-Michaud et al., 2004; Kotlowski et al., 2007; Palmela et al., 2018).

In this study, we used the honeybee model for the first time to explore the effects of human pathogenic *E. coli* LF82 on intestinal and cognitive function. We found that *E. coli* LF82 increased intestinal permeability by destroying the intestinal structure of honey-bees, causing manifestations similar to enteritis, which eventually led to a decrease in the lifespan of honeybees. Consistent with our findings, it was previously reported that *E. coli* LF82 infection induced loss of epithelial barrier function in mice (Carvalho et al., 2009; Mancini et al., 2021). Wine et al. also demonstrated that *E. coli* LF82 infection caused decreased transepithelial electrical resistance and increased macromolecular flux in human colonic cells by interrupting intercellular tight junctions (Wine et al., 2009). Thus, exposure to *E. coli* LF82 could impact host gut homeostasis. We also found the impairment of cognitive function in the *E. coli* LF82 group using an olfactory PER test, which reflected learning and memory abilities. These findings are in accordance with a previous report in which oral gavage of inflammatory *E. coli* isolated from the feces of mice with 2,4,6-trinitrobenzene sulphonic acid-induced colitis caused colitis as well as memory impairment (Jang et al., 2018). In another work, inflammatory *E. coli* isolated from the human gut caused colitis in mice, who also exhibited behaviors such as cognitive decline and depression during elevated plus maze, tail suspension, and forced swimming tasks (Kim et al., 2020). These results suggest that pathogenic *E. coli* may be involved in the cognitive impairment related to colitis.

The production and modification of metabolic, immunological, and neurochemical factors are well-known pathways of gut microorganisms signaling to the central nervous system (Kwong et al., 2017; Morais et al., 2021). In the present study, we found decreased levels of tryptophan metabolites, kynurenine and melatonin, in honeybees treated with *E. coli* LF82. Kynurenine is a major metabolite in the degradation of tryptophan, as well as the beginning of the kynurenine pathway. Lopatina et al. found that the reduction or deficiency of kynurenine, accomplished through gene mutation inactivating the first enzyme of tryptophan metabolism along the kynurenine pathway, tryptophan oxygenase, disturbed the storage of long-time memory in honeybees (Lopatina et al., 1995). Kynurenine is also the im-mediate metabolic precursor of kynurenic acid. The latter is an endogenous N-methyl-D-aspartate receptor antagonist, which plays an important role in synaptic plasticity linked to the development of learning and memory (Lynch, 2004). Carrillo-Mora et al. also demonstrated that L-kynurenine administration alleviated behavioral and morphological alterations induced by toxic soluble amyloid-beta (25–35) in rat hippocampus by increasing the level of kynurenic acid in the brain (Carrillo-Mora et al., 2010). As a well-known immune modulator, antioxidant, and neuroprotectant, melatonin is negatively correlated with brain inflammation and neurodegenerative disorders (Hardeland, 2010; Hardeland et al., 2011; Tordjman et al., 2017). In addition, melatonin has a vital role in neurogenesis from neural stem cells involved in neurogenesis as well as plasticity (Ramírez-Rodríguez et al., 2018). Therefore, the reduction of the above two neuroprotective metabolites may increase the susceptibility to brain disorders, and tryptophan metabolism might play a role in the enteritis-related cognitive impairment induced by *E. coli* LF82.

Like mammals and *Drosophila*, honeybees have conserved innate immunity against pathogens (Evans et al., 2006; Doublet et al., 2017). Previous studies demonstrated that gut pathogens, not attacking the brain directly, caused dramatical transcriptional changes in the brains of honeybees, including in genes involved in neural function and foraging behavior (Carvalho et al., 2009; McDonnell et al., 2013). Here, we found that the immune-related pathways such as the Toll and Imd signaling pathways, apoptosis, and the TNF signaling pathway are significantly upregulated in the brains of LF82-treated honeybees, consistent with previous research related to the honeybee infection model (Doublet et al., 2016). The pathways and specific mechanisms by which pathogens outside the brain activate neuroinflammation still need to be verified in future research (Coureuil et al., 2017; Li et al., 2019).

Apoptosis is a routine protective response to adverse stimuli such as inflammation, infection, and injury. However, excessive apoptosis of neurons can cause neurological disorders such as neurodegenerative diseases. In the present study, genes involved in apoptosis, such as *NF-κB2 p100* subunit and *caspase-1*, were up-regulated in LF82-treated honeybee brains. In mice, *p100* strings apoptosis through C-terminal death domain or IκB-like activity (Wang et al., 2002). The pro-apoptotic role of *p100* in the honeybee brain needs future experiments to verify. Caspase families are principal molecular components of the apoptosis program, and caspase-1 was upregulated along with the overlap of neuron apoptosis in the honeybee brain (Wu et al., 2015). Therefore, LF82 may cause excessive apoptosis of honeybee neurons by activating neuroinflammation, leading to cognitive impairment in bees.

In the past decade, most preclinical studies have begun to focus on gut microbes affecting brain functions, including cognition, and a broad theoretical system of the microbiota-gut-brain axis has been formed (Cryan et al., 2019). Being a new research field, the current aim is to elucidate underlying mechanisms primarily through germ-free, antibiotic-treated, transgenic or humanized mice and behavioral models (Chakrabarti et al., 2022). Models composed of organoids and co-cultured *in vitro* workflows are also used to study the gut-brain axis (Workman et al., 2017). In addition to the high cost, the complexity of mammalian models and organoids is an obstacle to accurately revealing exact mechanisms of the gut-brain axis. As a classic animal model for behavioral research, honeybees are beneficial in the study of micro-biota-gut-brain axis due to their low cost of feeding, simple composition of gut bacteria and easy manipulation, and simple neural composition (Menzel, 2009; Zheng et al., 2018).

In conclusion, this study presents a comprehensive analysis of the gut metabolism, brain transcriptome, and subsequent cognition sickness during human IBD-related pathogenic bacterial infection of honeybees. Further in-depth studies on the neuro-immune and metabolic pathways involved in regulating cognitive function are warranted; they will provide insight and understanding of the mechanisms of IBD-related bacteria inducing cognitive impairment. Meanwhile, our findings indicate that honeybees can serve as a potential animal model to explore fundamental mechanisms underlying the microbiota-gut-brain axis.

## Data availability statement

The datasets presented in this study can be found in online repositories. The names of the repository/repositories and accession number(s) can be found in the article/**Supplementary Material**.

## Author contributions

YY was responsible for the study idea, and outlined and revised the manuscript draft. RC completed the analysis of the experimental data and the original manuscript draft preparation. JC, ZZ, and YL conducted behavior experiments. KW provided the *E. coli* LF82 strain. YY and HZ obtained funding for this study. All authors contributed to the article and approved the submitted version.

## Acknowledgments

We would like to thank Xiaohuan Mu and Yan Zhang for their assistance with the analysis of the transcriptome data and uploading of the raw data.

## Conflict of interest

The authors declare that the research was conducted in the absence of any commercial or financial relationships that could be construed as a potential conflict of interest.

## Publisher’s note

All claims expressed in this article are solely those of the authors and do not necessarily represent those of their affiliated organizations, or those of the publisher, the editors and the reviewers. Any product that may be evaluated in this article, or claim that may be made by its manufacturer, is not guaranteed or endorsed by the publisher.

## Supplementary material

Supplementary Figure 1 Histopathologic evaluation with hematoxylin and eosin staining of the honeybee midgut in the different groups (Bars = 50 μm).

Supplementary Figure 2
**(A)** Up-regulated metabolites enriched in KEGG pathway in the LF82 group. **(B)** Enrichment pathways of metabolites upregulated in LF82 in the fecal metabolic pool of patients.

Supplementary Figure 3
**(A)** Volcano map of differentially expressed genes (DEGs) in the honeybee brain between LF82 and MG1655 (n = 3). **(B)** Representative enriched KEGG pathways downregulated in the LF82 group, compared with the MG1655 group.
