# Supplementary figures and images for: Inflammatory bowel disease-associated Escherichia coli strain LF82 in the damage of gut and cognition of honeybees
Source: Front Cell Infect Microbiol. 2022 Aug 25;12:983169. doi: 10.3389/fcimb.2022.983169 (PMC9453226; doi:10.3389/fcimb.2022.983169)

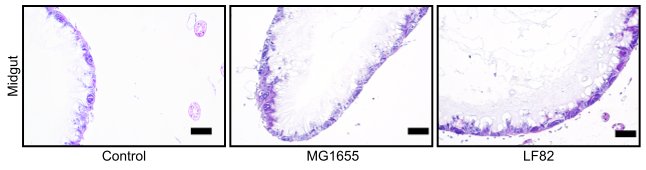

Supplement: Supplementary Figure 1 — Histopathologic evaluation with hematoxylin and eosin staining of the honeybee midgut in the different groups (Bars = 50 μm). [file Image_1.jpeg]

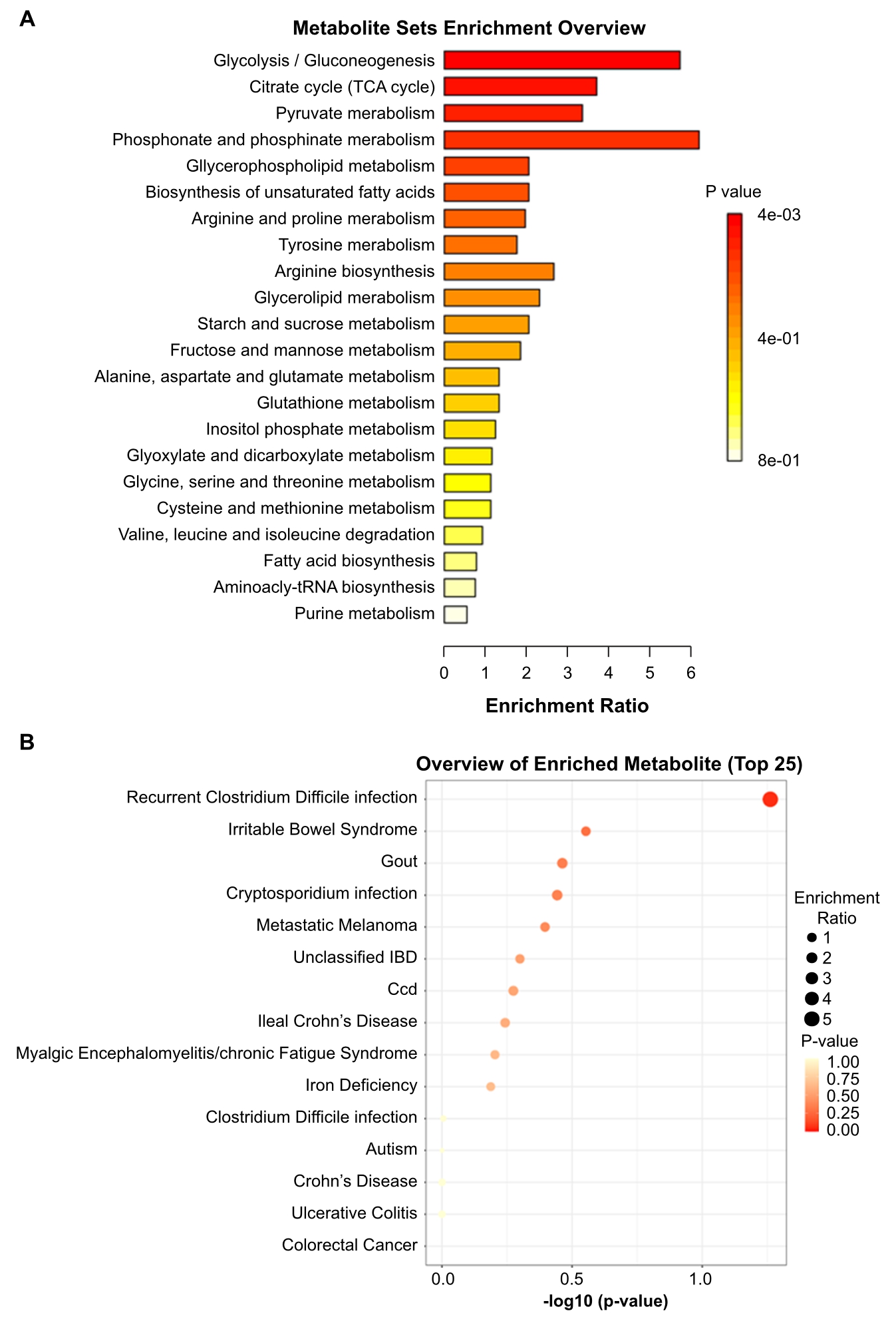

Supplement: Supplementary Figure 2 — (A) Up-regulated metabolites enriched in KEGG pathway in the LF82 group. (B) Enrichment pathways of metabolites upregulated in LF82 in the fecal metabolic pool of patients. [file Image_2.jpeg]

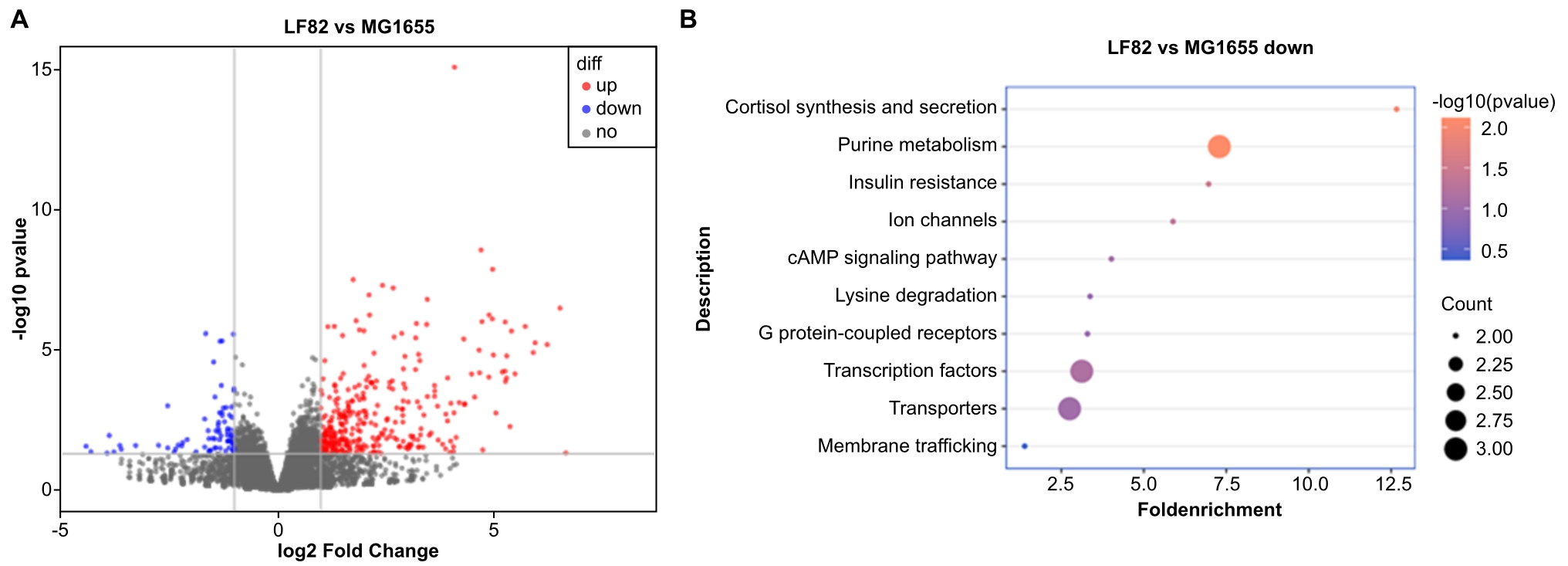

Supplement: Supplementary Figure 3 — (A) Volcano map of differentially expressed genes (DEGs) in the honeybee brain between LF82 and MG1655 (n = 3). (B) Representative enriched KEGG pathways downregulated in the LF82 group, compared with the MG1655 group. [file Image_3.jpeg]
